# Standortbestimmung der universitären Psychiatrie und Psychotherapie in Deutschland: Aufgaben und Herausforderungen: Ein Positionspapier der Lehrstuhlinhaber für Psychiatrie und Psychotherapie e. V. in Deutschland (LIPPs)

**DOI:** 10.1007/s00115-024-01688-4

**Published:** 2024-06-21

**Authors:** P. Falkai, T. Frodl, H. J. Grabe, R. Rupprecht, A. Philipsen

**Affiliations:** 1 Klinik für Psychiatrie und Psychotherapie, Ludwig-Maximilians-Universität München, Nußbaumstraße 7, 80366 München, Deutschland; 2 Klinik für Psychiatrie, Psychotherapie und Psychosomatik, Universitätsklinikum Aachen, Pauwelsstraße 30, 52074 Aachen, Deutschland; 3 Klinik für Psychiatrie und Psychotherapie, Universitätsmedizin Greifswald, Ellernholzstraße 1–2, 17489 Greifswald, Deutschland; 4 Klinik für Psychiatrie und Psychotherapie, Universität Regensburg, Universitätsstraße 84, 93053 Regensburg, Deutschland; 5 Klinik und Poliklinik für Psychiatrie und Psychotherapie, Universitätsklinikum Bonn, Venusberg-Campus 1, 53127 Bonn, Deutschland

## Einleitung

Die Rolle einer Universitätsmedizin besteht darin, eine Verbindung zwischen medizinischer Forschung, Lehre und Patientenversorgung herzustellen. Universitätsmedizinische Einrichtungen sind in der Regel an Universitäten angeschlossen und bieten eine breite Palette medizinischer Dienstleistungen an. Sie dienen zudem als Ausbildungsstätten für angehende Ärztinnen und Ärzte, Medizinstudierende und medizinisches Fachpersonal. Darüber hinaus sind Universitätsmedizinische Zentren oft führend in der medizinischen Forschung und Innovation, was zur Entwicklung neuer Behandlungsmethoden und medizinischer Technologien beiträgt.

Psychiatrisch-psychotherapeutische Universitätskliniken haben eine wichtige Rolle in der Erforschung, Diagnose, Behandlung und Prävention von psychischen Erkrankungen. Zu ihren Aufgaben gehören:

1. Ausbildung: Sie dienen als Ausbildungsstätten für angehende Psychiater, Psychologen und andere Fachkräfte im Bereich der psychischen Gesundheit. Studierende und Praktizierende können hier praktische Erfahrungen sammeln und von Experten lernen.
2. Forschung: Diese Kliniken führen innovative Forschung durch, um das Verständnis von psychischen Erkrankungen zu verbessern, neue Behandlungsmethoden zu entwickeln und Präventionsstrategien zu erforschen.
3. Patientenversorgung: Sie bieten spezifische und hochwertige psychiatrische Versorgung für Patienten mit verschiedenen psychischen Erkrankungen. Sie bieten Diagnose, Behandlung, Therapie und Unterstützung für Patienten und deren Familien.
4. Beratung und Prävention: Sie bieten Beratungsdienste, Präventionsprogramme und Unterstützung für die psychische Gesundheit der Gemeinschaft. Durch Aufklärung und Prävention können sie dazu beitragen, psychische Erkrankungen zu verhindern und das Bewusstsein für psychische Gesundheit zu stärken. Die Früherkennungszentren für Psychose sind ein wichtiger Meilenstein in diesem Bereich.

Die Versorgung psychiatrischer Erkrankungen an einer Universitätsklinik unterscheidet sich in mehreren Aspekten von der Versorgung an einer nichtuniversitären Versorgungsklinik:

1. Fachwissen und Expertise: Universitätskliniken verfügen in der Regel über ein breites Spektrum an Fachwissen und Expertise in der Psychiatrie. Sie sind führend in der psychischen Gesundheitsforschung und haben Zugang zu den neuesten Behandlungsmethoden und Innovationen.
2. Interdisziplinäre Expertenteams: An Universitätskliniken arbeiten interdisziplinäre Teams von Fachleuten zusammen, darunter Psychiater, Psychologen, Sozialarbeiter, Pflegekräfte und Therapeuten sowie eine umfassende kooperative Kollegschaft der weiteren medizinischen Bereiche mit Notfallmedizin, Innerer Medizin, Neurologie, Chirurgie sowie Intensivmedizin oder Palliativmedizin. Dies ermöglicht eine ganzheitliche und umfassende Versorgung der Patienten.
3. Ausbildung und Forschung: Universitätskliniken dienen auch als Ausbildungsstätten für angehende Psychiater und andere Fachkräfte im Bereich der psychischen Gesundheit. Die enge Verbindung zur Forschung trägt dazu bei, dass Patienten von aktuellen Erkenntnissen und Behandlungsmethoden profitieren.
4. Spezialisierung und Schwerpunkte: Universitätskliniken sind auf spezifische psychiatrische Erkrankungen, schwere Ausprägungen der Erkrankungen und spezifische Therapieansätze hochspezialisiert und bieten daher eine Vielzahl von spezialisierten Programmen und Behandlungsmöglichkeiten an.
5. Forschungsbasierte Behandlung: Die Behandlung an einer Universitätsklinik basiert auf evidenzbasierten Praktiken und häufig neuesten Forschungsergebnissen. Dies kann zu verbesserten Behandlungsergebnissen und innovativen Therapieansätzen führen.

Insgesamt bieten Universitätskliniken in der Psychiatrie eine hochwertige, umfassende und multidisziplinäre Versorgung, die von der Integration von Forschung, Lehre und Patientenversorgung profitiert. Seit der Gründung des ersten Lehrstuhls für Psychiatrie in Leipzig 1811 wurden weitere 41 Lehrstühle für Psychiatrie und Psychotherapie an deutschen medizinischen Fakultäten etabliert [1]. Diese hatten primär die Aufgabe, Medizinstudierenden die inhaltlichen Grundlagen des Fachs zu lehren und Ärzte und Ärztinnen fachärztlich weiterzubilden. Ganz nach dem Vorbild Humboldts wurden in der Folge auch in der Psychiatrie auf Basis der universitären wissenschaftlichen Tätigkeit die Diagnostik und Therapie von psychischen Erkrankungen wesentlich weiterentwickelt und verbessert. Im Laufe der vergangenen 200 Jahre haben die Lehrstühle für Psychiatrie und Psychotherapie immer zahlreichere Aufgaben innerhalb und außerhalb der medizinischen Fakultäten übernommen. Dies macht nun eine Standortbestimmung notwendig, zumal die Finanzierung dieser Aufgaben u. a. mit der Einführung der Richtlinie über die Ausstattung der stationären Einrichtungen der Psychiatrie und Psychosomatik (PPP-RL) und der anstehenden Krankenhausreform zunehmend schwieriger wird. Anhand von zentralen Thesen im Bereich Forschung, Lehre und Krankenversorgung werden die Aufgaben der universitären Psychiatrie und Psychotherapie in Deutschland im Folgenden herausgearbeitet und Forderungen für die Zukunft an das Fach, aber auch an Politik und Gesellschaft gestellt.

## 1. Die Rolle der universitären Psychiatrie und Psychotherapie in Deutschland in der Forschung

### 1. Die deutsche Psychiatrie und Psychotherapie belegt mit ihrem Forschungsoutput Spitzenplätze

Die universitäre psychiatrisch-psychotherapeutische Forschung in Deutschland nimmt hinsichtlich der wissenschaftlichen Produktivität – oder gemessen an der Anzahl der Publikationen – weltweit mit Platz 3 einen Spitzenplatz hinter den USA und Großbritannien ein [2]. Nach einer Evaluierung der Anzahl der Publikationen pro Land befindet sich Deutschland für die Diagnosen Alkoholabhängigkeit, Schizophrenie und Depression auch in diesen diagnosespezifischen Auswertungen auf Platz 2 bzw. 3, zusammen mit den USA, Großbritannien und Kanada. Eine Korrektur für die Größe der Bevölkerung wurde nicht vorgenommen [3].

### 2. Bei der Forschungsförderung liegt die deutsche Psychiatrie und Psychotherapie im unteren Zehntel der medizinischen Disziplinen

Für die Förderung der Erforschung psychischer Erkrankungen gibt das BMBF jährlich 20,2 Mio. € aus, die DFG ca. 13,4 Mio. € pro Jahr (Medizin DFG insgesamt: 749 Mio. €, womit der Anteil für die Psychiatrie 1,8 % beträgt). Der G‑BA Innovationsfonds Versorgungsforschung wendet ca. 21 Mio. € auf und der G‑BA Innovationsfonds Neue Versorgungsformen jährlich ca. 38,2 Mio. € und für Leitlinien nochmals 3 Mio. €, ebenfalls jeweils pro Jahr. Somit wird die Erforschung von psychischen Erkrankungen in Deutschland jährlich mit insgesamt 95,8 Mio. € gefördert [4–7]. Betrachtet man diese Investitionen im Verhältnis zur gesellschaftlichen Relevanz, so entsprechen diese 95,8 Mio. € p. a. lediglich 0,09 % der Gesamtausgaben für Wissenschaft, Forschung und Entwicklung in Deutschland (2020: 105 Mrd. € bzw. 0,0002 % des Bundeshaushalts; 2023 ca. 420 Mrd. €; [4]). Die direkten Kosten, also Kosten, die im Gesundheitswesen unmittelbar aufgrund psychischer Erkrankungen entstehen, beliefen sich im Jahr 2020 in Deutschland auf rund 56,4 Mrd. € und sind in den vergangenen Jahren deutlich angestiegen. Psychische Erkrankungen machen 13 % der gesamten Krankheitskosten aus. Nur Krankheiten des Kreislaufsystems verursachen höhere Kosten [7].

### 3. Klinisch-translationale Forschung bei psychischen Erkrankungen findet überwiegend in Universitätskliniken für Psychiatrie und Psychotherapie statt

Als prägnantes Beispiel für die Forschungsstärke der deutschen Lehrstühle und die Diskrepanz zu den zur Verfügung gestellten Forschungsmitteln sollen exemplarisch die bipolaren Störungen dienen. Bipolare Störungen beginnen früh im Leben (für die Mehrzahl der Betroffenen zwischen dem 15. und 25. Lebensjahr) und bestehen in der Regel lebenslang. Sie nehmen den 5. Platz bei den *„disability-adjusted life years“* (DALY) ein, haben eine Prävalenz von ca. 1 % in der Erwachsenenbevölkerung und verursachen jährliche medizinische Kosten von 2,5 Mrd. € in Deutschland. In den letzten 10 Jahren wurden auf diesem Gebiet 1415 Originalarbeiten und 348 Reviews unter Beteiligung von Autoren aus Deutschland publiziert, davon war in 52 % der Artikel der korrespondierende Autor aus Deutschland. Betrachtet man die produktivsten deutschen Autoren, so arbeiten diese überwiegend in psychiatrischen Universitätskliniken, mit Ausnahme derer aus dem Institut für Humangenetik in Bonn, dem Zentralinstitut für seelische Gesundheit (ZI) in Mannheim (das universitätsmedizinische Aufgaben mit der medizinischen Fakultät Mannheim der Universität Heidelberg übernimmt) und dem MPI in München. Hinsichtlich der nationalen und internationalen Förderung für den Bereich der bipolaren Störungen beträgt die Gesamtsumme lediglich 1,4 Mio. € [8], was in einer deutlichen Diskrepanz zur Relevanz dieser schweren und häufigen psychischen Erkrankung steht.

Im Hinblick auf die Anwendung neuer Forschungsergebnisse, besonders im Bereich Diagnostik und Therapie, sind die psychiatrisch-psychotherapeutischen Universitätskliniken die entscheidenden Institutionen, sowohl in der Breite als auch in der Tiefe. Im Hinblick auf neue pharmakologische Therapiestrategien (in den letzten Jahren bspw. die Implementation der Esketamin-Therapie in die klinische Routine, aber auch andere schnell wirkende Antidepressiva wie Psychedelika) oder neurostimulatorische Verfahren waren und sind Universitätskliniken die Motoren des Fortschritts; so war lange Zeit die transkranielle Magnetstimulation (rTMS) ganz überwiegend nur an Unikliniken verfügbar, was momentan bspw. für die Vagusnervstimulation (VNS) gilt. Psychotherapeutische Verfahren werden zwar auch an psychologischen Instituten evaluiert, jedoch ist die hier behandelte Patientenklientel deutlich leichter erkrankt. Insofern sind psychiatrische Unikliniken entscheidend in der Entwicklung, Evaluierung und Implementation neuer psychotherapeutischer Interventionen gerade für schwer, akut und/oder komplex erkrankte Patienten. Im Bereich der Alzheimer-Krankheit haben universitäre psychiatrisch-psychotherapeutische Kliniken die biomarkergestützte Frühdiagnostik über mehr als 20 Jahre wissenschaftlich und klinisch geprägt. Ein wesentlicher Teil aller Zentren für klinische Studien im Bereich der Alzheimer-Krankheit ist an universitären psychiatrischen Gedächtnisambulanzen angesiedelt, die aktuell als Expertenzentren auch die Einführung molekularer krankheitsmodifizierender Therapie vorbereiten.

Betrachtet man andere Forschungsinstitutionen im Bereich der Grundlagenwissenschaften (z. B. MPG, Leibniz, Helmholtz, Fraunhofer), der Psychologie oder außeruniversitärer Zentren, so ist der Beitrag zur klinischen Translation fokussiert auf sehr wenige, einzelne Themen oder aber gar nicht vorhanden. Darüber hinaus hatte sich die pharmazeutische Industrie in den letzten Jahrzehnten weltweit aus dem Bereich psychischer Erkrankungen zurückgezogen, insbesondere auch aus dem Standort Deutschland. Weitere Gründe für den Rückzug aus Deutschland sind das sehr strenge Arzneimittelgesetz (z. B. im Vergleich zu Spanien oder den NL) und v. a. das AMNOG-Verfahren. Erst in den letzten Jahren gibt es nun wieder ein vermehrtes Interesse und Aktivitäten in diesem Bereich, was durch eine steigende Zahl von Start-ups, Biotechfirmen und Medtech-Firmen gut ergänzt wird. Insbesondere im Bereich der Behandlung depressiver Erkrankungen trugen deutsche Universitätskliniken wesentlich zur Rückkehr der Industrie in die ZNS-Forschung bei. Die Erforschung eines gestörten glutamatergen Stoffwechsels, zum Beispiel an den Universitäten Jena, München und Tübingen, führte zusammen mit einer schnellen Übertragung im Rahmen von „investigator-initiated trials“ (IIT) in den letzten 15 Jahren zu einer pathophysiologischen und therapeutischen neuen Ära in der Behandlung mit glutamaterg wirksamen Substanzen oder Hirnstimulationsverfahren.

#### Zusammenfassung 1.

Universitätskliniken für Psychiatrie und Psychotherapie sind und bleiben die zentrale Säule für die Durchführung translational-klinischer Studien, insbesondere industrieunabhängige, durch Wissenschaftler initiierte Studien. Diese sind die Grundlage der modernen evidenzbasierten Medizin. Nur durch deren Beitrag können zudem die „Todestäler der Translation“ zwischen Grundlagen und klinischer Forschung überwunden werden und die Forschung anschließend in die Anwendung gelangen [9]. Nur sie gewährleisten klinische Forschung in der Tiefe und Breite des gesamten Spektrums psychischer Erkrankungen. Sie sind der Ort, wo ärztlich-medizinische Forschung möglich ist und gefördert werden muss. Sie erlauben ein hohes Maß an interdisziplinärer Zusammenarbeit sowohl innerhalb der Medizin, z. B. mit der Neurologie, als auch außerhalb, z. B. mit der Psychologie. Sie gewährleisten eine kontinuierliche Gewinnung von wissenschaftlichen Nachwuchskräften aus dem Kreis der Medizinstudierenden, was ohne ein klares translationales Forschungsprofil nicht möglich wäre. Über die Landeszuführungsbeträge für Forschung und Lehre, ergänzt durch die leistungsorientierte Mittelvergabe (LOM), wird eine Grundausstattung für die Durchführung von Wissenschaft an den Universitäten gewährleistet; ansonsten finanziert sich diese über die Einwerbung von Drittmitteln. Ein erfolgreiches Beispiel für die Zusammenarbeit psychiatrischer Universitätskliniken und außeruniversitärer Forschungseinrichten in der Psychiatrie in Deutschland stellt das BMBF-geförderte Forschungsnetz zu psychischen Erkrankungen dar. Dieser Ansatz wird derzeit auch im Deutschen Zentrum für Psychische Gesundheit (DZPG) weiterentwickelt [10].

## 2. Rolle der universitären Psychiatrie und Psychotherapie in Deutschland in der Aus- und Weiterbildung

Zum Wintersemester 2022/23 wurden deutschlandweit 108.130 Studierende der Humanmedizin gezählt [11], wovon jedoch nur die Hälfte direkt in der klinischen Medizin berufstätig wird. Die andere Hälfte orientiert sich im Ausland oder wird in Deutschland in der Industrie oder im Verwaltungsbereich tätig. In Deutschland arbeiten insgesamt 14.625 Fachärztinnen und Fachärzte für Psychiatrie und Psychotherapie bzw. Nervenheilkunde; im Jahr 2022 wurden 555 neue Facharzttitel für Psychiatrie und Psychotherapie anerkannt [7]. Die fachärztliche Versorgung außerhalb der Ballungszentren ist nach wie vor schwierig, und es zeigt sich kein Trend zur Verbesserung dieser Situation. Im Gegenteil ist eine weitere Verschlechterung der ambulanten psychiatrischen Versorgung zu erwarten, da ein großer Anteil der niedergelassenen Psychiater sich dem Rentenalter annähert, während die Inanspruchnahme psychiatrisch-psychotherapeutischer Behandlung zunimmt.

### 1. Lehre in der Psychiatrie und Psychotherapie ist stark interdisziplinär ausgerichtet

Universitätskliniken für Psychiatrie und Psychotherapie sind Teile der jeweiligen Universitätsmedizin und somit auch Bestandteil der fachübergreifenden und interdisziplinären Lehre. Hierzu gehören gemeinsame oder aufeinander abgestimmte Lehrveranstaltungen wie z. B. mit der medizinischen Psychologie, der psychosomatischen Medizin, der Neurologie, der Altersmedizin, der Notfallmedizin, der Rechtsmedizin, den Grundlagenfächern der Gesundheitswissenschaften (Anatomie, Physiologie, Pharmakologie) oder anderen Fachgebieten wie z. B. der Psychologie.

### 2. Aus- und Weiterbildung in der Psychiatrie und Psychotherapie sichern die kontinuierliche Gewinnung von Nachwuchskräften

Insbesondere die Ausbildung von Medizinstudierenden bietet eine sehr gute Möglichkeit, Interesse für das Fach Psychiatrie und Psychotherapie zu wecken und somit den fachärztlichen Bestand stabil zu halten oder optimalerweise sogar zu erhöhen. Psychiatrische Universitätskliniken sind hierfür prädestiniert, da sie nicht nur ein umfassendes, multidisziplinäres Lehrangebot vorhalten, sondern zudem eine Verbindung zu aktuellen wissenschaftlichen Ergebnissen gewährleisten, was die Lehre interessant und abwechslungsreich macht und eine evidenzbasierte Versorgung sichert. Zudem obliegt es den psychiatrischen Universitätskliniken, auf dem Wege der Lehrforschung neue Lehrformate zu entwickeln, die die Entwicklungen im Fach, z. B. den trialogischen Gedanken, aufgreifen. So werden in jüngster Zeit über die üblichen Patientenvorstellungen hinaus in trialogischen Veranstaltungen lebensnah die Bedürfnisse psychisch erkrankter Menschen und ihrer Familien anschaulich gemacht und das Stigmaproblem von Menschen mit psychischen Erkrankungen behandelt. Wenn wie an vielen Standorten die psychiatrische Lehre jedoch am Ende des Medizinstudiums (z. B. im 10. Semester) angesiedelt wird, gestaltet sich u. a. die Vergabe von Doktorarbeiten bzw. Qualifizierungsarbeiten an interessierte und leistungsfähige Medizinstudierende schwierig. Eine stärkere Integration der Forschung im Rahmen einer durchzuführenden Seminararbeit im letzten Studienabschnitt ist hilfreich, aber nicht ausreichend.

Die Forderung der DFG, ca. 10 % der Weiterbildungsplätze für Clinician Scientists zu reservieren, ist sehr zu unterstützen: In diesem Forschungs-Track wird den angehenden Fachärztinnen und -ärzten die Möglichkeit gegeben, neben einer profunden klinischen Ausbildung auch eine ebenso profunde wissenschaftliche Ausbildung, wie z. B. in molekularen oder bildgebungsbasierten Techniken, zu erlangen. Dass dies möglich ist, zeigen viele von den Fakultäten selbst oder beispielweise der DFG oder privaten Stiftungen wie der Else Kröner-Fresenius-Stiftung finanzierte Clinician-Scientist-Programme. Solche Programme sind beispielsweise in den Niederlanden bereits etabliert und bringen einen klinisch wie wissenschaftlich sehr gut qualifizierten Nachwuchs hervor. Da viele dieser Programme nur temporär zur Verfügung stehen, sollte darauf gedrängt werden, dass diese ein strukturierter Teil der Aus- und Weiterbildung an Universitätskliniken werden.

### 3. Lehre in der Psychiatrie ist durch substanziellen Lehrexport und Lehrlast geprägt

Universitätskliniken für Psychiatrie und Psychotherapie gewährleisten die studentische Ausbildung im Fach Psychiatrie und Psychotherapie, wozu die Gewährleistung von Kapazitäten für das Praktische Jahr (PJ) und Famulaturen gehört. Darüber hinaus ermöglichen sie die Weiterbildung einer Vielzahl von Ärztinnen und Ärzten in Weiterbildung und garantieren damit die Aufrechterhaltung der Versorgung von Menschen mit psychischen Erkrankungen. Sie unterstützen aber nicht nur die Lehre im Fach Psychiatrie und Psychotherapie, sondern leisten Lehrexport z. B. in die Psychologie, psychosomatische Medizin, Allgemeinmedizin, Medizinpsychologie, Jura, Biologie, Gesundheitswissenschaften und Pharmakologie. Das „Direktstudium Psychotherapie“ erfordert für den Bachelor, aber insbesondere für den Master eine substanzielle klinische Unterstützung durch psychiatrische Universitätskliniken. Nur an den Universitätskliniken kann eine hochqualitative Ausbildung in klinischen Fragestellungen erfolgen. Neben der Gewährleistung einer Forschungsinfrastruktur soll der Landeszuführungsbetrag auch die Lehrleistung adäquat finanzieren. Hier tut sich wegen der Ausweitung von Lehrleistung, z. B. zur Unterstützung des Direktstudiengangs Psychotherapie, ein zunehmendes Defizit auf.

#### Zusammenfassung 2.

Die Lehre in der Psychiatrie und Psychotherapie wird entscheidend von den Universitätskliniken für Psychiatrie und Psychotherapie geleistet. Hier ergibt sich die Möglichkeit, Lehre und Forschung miteinander zu verknüpfen und damit diese interessant und aktuell zu gestalten. Leider erfolgt die Ausbildung von Medizinstudierenden an zahlreichen Standorten zu einem sehr späten Zeitpunkt während des Studiums, was z. B. die Übernahme einer Promotionsarbeit bzw. Qualifizierungsarbeit in diesem Bereich erschwert. Die Lehrlast für Universitätskliniken für Psychiatrie und Psychotherapie hat u. a. wegen der Übernahme von Lehrtätigkeiten im Rahmen des Direktstudiengangs Psychotherapie zugenommen, und die Kosten für die Lehre können nicht mehr vollumfänglich über die Landeszuführungsbeträge ergänzt und durch LOM gedeckt werden. Clinician-Scientist-Programme sind eine essenzielle Möglichkeit zur Förderung des wissenschaftlichen Nachwuchses. Entsprechende Programme müssen an allen Fakultäten etabliert werden und auch Plätze für Nachwuchskräfte aus Psychiatrie und Psychotherapie vorhalten.

## 3. Rolle der universitären Psychiatrie in Deutschland in der Versorgung

**Abb. 1:**
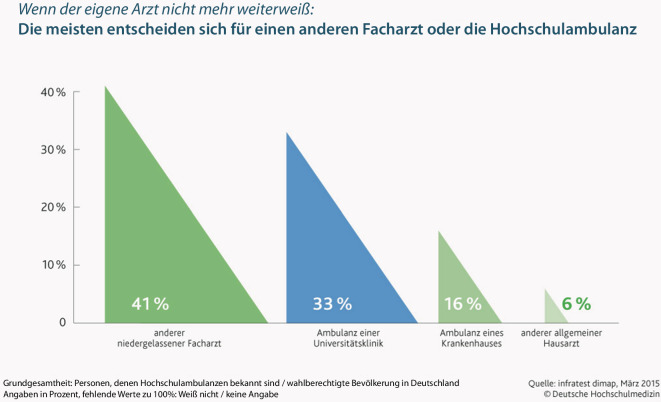
Universitätskliniken in der ambulanten Versorgung

Stationäre, teilstationäre und ambulante Einrichtungen der Psychiatrie und Psychotherapie tragen einen substanziellen Teil der Versorgung von Menschen mit psychischen Erkrankungen in Deutschland. Wie eingangs erwähnt ist die klinische Versorgung durch PPRRL nicht bedarfsgerecht gesichert, gerade Universitätskliniken sollten jedoch eine wichtige Rolle bei der wissenschaftlich fundierten Bemessung von Versorgungsbedarf spielen. Die Universitätskliniken übernehmen ca. 33 % der ambulanten Versorgung (Abb. 1; [12]), und müssen zusätzlich ihre Hauptaufgaben von Forschung und Lehre bewältigen. Es ist somit von zentraler Bedeutung, dass personelle Kapazitäten psychiatrischer Universitätskliniken ausgebaut werden. Darüber hinaus müssen jedoch Mechanismen vorhanden sein, damit sie auch alle anderen, zuvor genannten, Aufgaben bewältigen können. Die strukturellen zusätzlichen Anforderungen im universitätsklinischen Bereich sollten auch im Bereich der Psychiatrie und Psychotherapie zusätzlich vergütet werden, wie im Bereich der somatischen Medizin (Level IIIu) von der Krankenhauskommission vorgeschlagen. Zu diesen zentralen Themen der Versorgung positioniert sich die DGPPN ebenfalls eindeutig und fordert dies ausdrücklich.

### 1. Psychiatrische Universitätskliniken sichern Aus‑, Weiter- und Fortbildung

Mit ihren stationären Betten, tagesklinischen Plätzen, stationsäquivalenten Behandlungsangeboten und Hochschul- und Institutsambulanzen ermöglichen psychiatrische Universitätskliniken die studentische *Ausbildung* sowie die ärztliche *Weiterbildung *und *fachärztliche Fortbildung *und tragen somit wesentlich zur Versorgung der Bevölkerung bezüglich psychischer Gesundheit und Krankheit bei. In vielen deutschen psychiatrischen Kliniken muss bereits ärztliches Personal aus dem Ausland gewonnen werden, was gerade in der psychiatrischen Versorgung aufgrund der sprachlichen Barrieren mit besonderen Herausforderungen einhergeht. Hinzu kommt, dass mittel- und langfristig die medizinische Versorgung – insbesondere in ländlichen Gebieten – nicht mehr aufrechterhalten werden kann.

### 2. Psychiatrische Universitätskliniken sichern die interdisziplinäre (Notfall‑)Versorgung

Psychiatrisch-psychotherapeutische Universitätskliniken sichern die *interdisziplinäre (Notfall‑)Versorgung* sowohl von Menschen mit psychischen Erkrankungen und somatischer Komorbidität als auch von somatisch Erkrankten mit psychischer Komorbidität ab. Über Konsil- und Liaisondienste kann eine psychiatrisch-psychotherapeutische Versorgung auch bei Patienten mit komplexen oder seltenen Erkrankungen bis hin zu Transplantationspatienten ermöglicht werden. Beispiele für die interdisziplinäre Versorgung sind zum einen neurodegenerative Erkrankungen mit Bewegungsstörungen und psychischen Symptomen, die gemeinsam mit der Neurologie behandelt werden. Zum anderen Suchterkrankungen, für deren Versorgung eine enge Verzahnung von psychiatrisch-psychotherapeutischer Kompetenz und Innerer Medizin notwendig ist, um sowohl die körperliche Entgiftung als auch die Behandlung der Grunderkrankung der Abhängigkeit zu gewährleisten. Fehlt die Integration psychiatrischer Kompetenz in die Somatik, so ist der dadurch entstehende Drehtüreffekt gut belegt [13], welcher auf einer unzureichenden Erkennung und Behandlung psychischer Komorbiditäten beruht. Depressionen gelten als wichtiger unabhängiger Risikofaktor für Herz-Kreislauf-Erkrankungen und als prognostisch ungünstig bei akutem Herzinfarkt. Psychiatrische Universitätskliniken behandeln diese Komorbiditäten über Konsil- und Liaisondienste. Ein relevanter Anteil der Krankenhausaufnahmen entfällt auf Alkoholfolgeprobleme, wie HNO-Tumoren, Leberzirrhose oder Frakturen; die hierfür ursächlichen Suchtstörungen werden von psychiatrischen Kliniken behandelt.

### 3. Psychiatrische Universitätskliniken ermöglichen klinisch-translationale Forschung

Ohne die Versorgungsstruktur von psychiatrischen Universitätskliniken ist eine klinisch *translationale Forschung *von den Grundlagen in die Klinik und zurück nicht denkbar, denn sie wird, wie oben ausgeführt, auch primär dort realisiert. An psychiatrischen Universitätskliniken werden neue Therapien entwickelt, eingesetzt, evaluiert und in die Dissemination gebracht; Gleiches gilt für die Prävention. Hierzu gehört auch eine Integration von neuen Versorgungskonzepten wie Früherkennungs- und Interventionseinheiten.

#### Zusammenfassung 3.

Psychiatrisch-psychotherapeutische Universitätskliniken nehmen an der Versorgung von Menschen mit psychischen Erkrankungen teil, brauchen jedoch gleichzeitig eine Versorgungsstruktur aus stationären Betten, stationsäquivalenten, tagesklinischen und ambulanten Plätzen, um ihren Lehrverpflichtungen nachzukommen und Medizinstudierende vollumfänglich aus- und Assistenzärztinnen und -ärzte weiterzubilden. Darüber hinaus ist die Versorgungsstruktur Garant für eine dringend notwendige translationale Forschung, welche in Deutschland im Wesentlichen durch die Universitätskliniken gewährleistet wird.

## 4. Forderungen für die Weiterentwicklung der Psychiatrie und somit der psychiatrisch-psychotherapeutischen Universitätskliniken

Psychiatrisch-psychotherapeutische Universitätskliniken unterliegen einem inhaltlichen und finanziellen Wettbewerb mit anderen Fächern innerhalb und außerhalb der Medizin sowie gegenüber außeruniversitären psychiatrischen Kliniken. Damit sie zukünftig ihre Aufgaben besser erfüllen können, werden folgende Forderungen an das Fach selber, die Politik und Gesellschaft gestellt:

### 1. Die Psychiatrie ist eine medizinische Disziplin und muss sich als solche weiterentwickeln

Die Psychiatrie und Psychotherapie ist eine Disziplin der Medizin und muss daher auch ihre Aufgaben in der Diagnostik und Therapie psychischer Erkrankungen vollumfänglich erfüllen. Sie sollte realisieren, dass die Reliabilität ihrer Diagnosen vergleichbar mit den anderen Disziplinen und lediglich ihre Validität schlechter ist [14]. Grund für die aktuell noch unzureichende Validität ist ein fehlendes pathophysiologisches Verständnis psychischer Erkrankungen. Hier besteht die langjährige Forderung [15], dass auch das Fach Psychiatrie und Psychotherapie investieren und eine Roadmap verfolgen muss, damit dies in den nächsten Jahrzehnten signifikant verbessert werden kann. So hat die Neurologie vor mehreren Jahrzehnten in die Grundlagen investiert und kann heute im Bereich der multiplen Sklerose die Früchte von pathophysiologisch orientierten Therapien und prognostischen Markern für Patientensubgruppen ernten. Die universitäre Gerontopsychiatrie ist zusammen mit der Neurologie und Geriatrie international unter den führenden Arbeitsgruppen im Bereich der Früh- und Differenzialdiagnostik der Demenz bei Alzheimer-Krankheit. Hier haben Biomarker im lumbalen Liquor Einzug in die gemeinsamen S3-Demenzleitlinien der neuropsychiatrischen Fachgesellschaften genommen (www.dgppn.de), und eine blutbasierte Demenzdiagnostik für die Routinediagnostik ist absehbar. Die drohende Alzheimer-Demenz kann mit hoher diagnostischer Sicherheit bereits mehr als 10 Jahre vor dem Demenzsyndrom blut- und liquorbasiert präklinisch identifiziert werden [16], was besonders relevant für den sekundärpräventiven Einsatz innovativer antikörperbasierter Therapien sein wird [17, 18], die nach Zulassung in den USA (mAb Lecanemab) voraussichtlich auch zeitnah in Europa verfügbar sein werden. Hier werden die universitären gerontopsychiatrischen Gedächtnisambulanzen zukünftig eine wichtige Rolle in der biomarkergestützten Demenzdiagnostik und medikamentös präventiven Behandlung übernehmen.

### 2. Psychiatrische Forschung und Lehre müssen besser finanziert werden

Die finanziellen Mittel, die der Psychiatrie für die Forschung zur Verfügung stehen, sind gemessen an der volkswirtschaftlichen Bedeutung psychischer Erkrankungen deutlich zu gering und sollten gestaffelt über 10 Jahre auf inflationsbereinigt mindestens das Doppelte, d. h. 180 Mio. € jährlich, gesteigert werden [19]. Zusätzliche finanzielle Mittel könnten eine frühzeitige umfassende und einheitliche Diagnostik und deren Weiterentwicklung auf der Grundlage von Klinik und computergestützten Biomarkern am Beginn von psychiatrischen Erkrankungen, die Entwicklung spezifischer individualisierter Therapien sowie die Behandlung von Schwerstkranken mit Entwicklung und Implementierung von Komplexprogrammen sichern. Dies kann jedoch nicht ausschließlich ein Prozess von außen sein, sondern muss weiterhin von Anstrengungen des Fachs begleitet sein, z. B. mit der Einwerbung von EU-Förderung und SFB mit dem Schwerpunkt der Erforschung der Pathophysiologie und innovativer Therapieverfahren psychischer Erkrankungen. Neben der Forschung steht auch die psychiatrische Lehre zunehmend finanziell unter Druck. Bei der steigenden Lehrlast müssen Fakultäten und die für sie verantwortlichen Ministerien prüfen, wie der Lehrexport, z. B. für den Aufbau des Direktstudiengangs Psychotherapie, zusätzlich finanziert werden kann. Die bereits erwähnten Clinician-Scientist-Programme sind eine essenzielle Möglichkeit zur Förderung des wissenschaftlichen Nachwuchses, weshalb entsprechende Programme auch für Nachwuchskräfte aus Psychiatrie und Psychotherapie finanziell gefördert werden müssen.

### 3. Psychische Erkrankungen müssen somatischen in allen Belangen gleichgestellt werden

Es gibt eine ganze Anzahl von Umweltrisikofaktoren wie Armut, Klimaveränderungen oder kriegerische Konflikte und zukünftige Herausforderungen wie den demografischen Wandel, die gleichermaßen Einfluss sowohl auf die somatische als auch auf die psychische Gesundheit von Menschen haben. Ein Risikofaktor, welcher jedoch insbesondere psychische Erkrankungen betrifft, sind die mit diesen verbundenen Stigmata. Untersuchungen haben gezeigt, dass die Stigmatisierung bei affektiven Erkrankungen in den letzten zwei Jahrzehnten rückläufig war, dies jedoch nicht für Sucht- oder psychotische Erkrankungen oder Demenzen gilt. Die Stigmatisierung von Menschen mit psychischen Erkrankungen ist nicht nur in der Gesellschaft allgemein, sondern auch in der Medizin weiterhin präsent; hier kommt psychiatrischer Lehre eine herausragende Bedeutung zu, die neue Lehrformate erfordert, die die persönliche Begegnung mit den Schicksalen Betroffener intensivieren. Solche aber müssen nicht nur entwickelt, sondern auch systematisch evaluiert werden. Politik und Gesellschaft sind aufgerufen, gemeinsam mit der Psychiatrie über psychische Erkrankungen zu informieren und auf eine Gleichstellung von psychischen und somatischen Erkrankungen abzuzielen. Eine Möglichkeit wäre, neben einem festen Anteil von Arbeitsplätzen für Menschen mit körperlichen Behinderungen den gleichen Anteil auch für Menschen mit einer psychischen Erkrankung vorzuhalten. Eine feste, bezahlte Arbeit ist für Menschen mit psychischen Erkrankungen der beste Prädiktor für die Verhinderung eines Rezidivs.

### 4. Viersäulenmodell der psychiatrischen Universitätsmedizin

Die Pandemie hat gezeigt, dass der Universitätsmedizin eine Schlüsselrolle zukommt, auf Krisen agil und adaptiv zu reagieren. Auf die psychiatrische Universitätsmedizin übertragen hat diese nicht nur traditionelle Aufgaben in Forschung, Lehre und Krankenversorgung, sondern zusätzlich auch koordinierende und konzeptionelle System- und Zukunftsaufgaben gerade im Hinblick auf vulnerable Gruppen (sog. Viersäulenmodell). Aus dieser Verantwortung resultiert ein finanzieller Mehrbedarf (z. B. für Digitalisierung; [20]).

## Fazit für die Praxis

Hinsichtlich ihrer Rolle in der Forschung belegt die deutsche Psychiatrie mit ihrer Forschungsleistung national und international Spitzenplätze, bei der Forschungsförderung liegt sie jedoch lediglich im unteren Zehntel der medizinischen Disziplinen, obwohl die klinisch-translationale Forschung bei psychischen Erkrankungen überwiegend in psychiatrischen Universitätskliniken stattfindet. Diese Schere zwischen hoher Leistung und Unterfinanzierung ist unter den aktuellen o. g. Herausforderungen nicht aufrechtzuerhalten. Was die Rolle der Lehre in der universitären Psychiatrie und Psychotherapie betrifft, so ist diese durch ihre stark interdisziplinäre Ausrichtung und somit gleichzeitig auch durch einen substanziellen Lehrexport und eine entsprechend hohe Lehrlast geprägt. Hinsichtlich der Versorgung sichern psychiatrische Universitätskliniken die Aus‑, Weiter- und Fortbildung, sichern die interdisziplinäre (Notfall‑)Versorgung und ermöglichen auch hier die klinisch-translationale Forschung.

Um zukünftig ihre Aufgaben besser erfüllen zu können, werden folgende Forderungen an das Fach Psychiatrie und Psychotherapie selber sowie an Politik und Gesellschaft gestellt: Die Psychiatrie und Psychotherapie als medizinische Disziplin mit hoher gesellschaftlicher und gesundheitsökonomischer Relevanz muss als solche deutlich sichtbarer werden, psychiatrische Forschung und Lehre müssen besser finanziert und psychische Erkrankungen den somatischen in allen Belangen gleichgestellt werden.
